# pH-Responsive Poly(ethylene glycol)-*b*-poly(2-vinylpyridine) Micelles for the Triggered Release of Therapeutics

**DOI:** 10.3390/pharmaceutics15030977

**Published:** 2023-03-18

**Authors:** Kyle Brewer, Fengxiang Bai, Anton Blencowe

**Affiliations:** Applied Chemistry and Translational Biomaterials (ACTB) Group, Centre for Pharmaceutical Innovation (CPI), UniSA Clinical and Health Sciences, University of South Australia, Adelaide, SA 5000, Australia; brekr002@mymail.unisa.edu.au (K.B.); fengxiang.bai@student.adelaide.edu.au (F.B.)

**Keywords:** pH-responsive, micelle, poly(ethylene glycol), poly(2-vinylpyridine), doxorubicin, gossypol, CDKI-73, EPR effect, triggered release, photo-RAFT

## Abstract

The use of pH-responsive polymeric micelles is a promising approach to afford the targeted, pH-mediated delivery of hydrophobic drugs within the low-pH tumour milieu and intracellular organelles of cancer cells. However, even for a common pH-responsive polymeric micelle system—e.g., those utilising poly(ethylene glycol)-*b*-poly(2-vinylpyridine) (PEG-*b*-PVP) diblock copolymers—there is a lack of available data describing the compatibility of hydrophobic drugs, as well as the relationships between copolymer microstructure and drug compatibility. Furthermore, synthesis of the constituent pH-responsive copolymers generally requires complex temperature control or degassing procedures that limit their accessibility. Herein we report the facile synthesis of a series of diblock copolymers via visible-light-mediated photocontrolled reversible addition-fragmentation chain-transfer polymerisation, with a constant PEG block length (90 repeat units (RUs)) and varying PVP block lengths (46–235 RUs). All copolymers exhibited narrow dispersity values (*Đ* ≤ 1.23) and formed polymeric micelles with low polydispersity index (PDI) values (typically <0.20) at physiological pH (7.4), within a suitable size range for passive tumour targeting (<130 nm). The encapsulation and release of three hydrophobic drugs (cyclin-dependent kinase inhibitor (CDKI)-73, gossypol, and doxorubicin) were investigated in vitro at pH 7.4–4.5 to simulate drug release within the tumour milieu and cancer cell endosome. Marked differences in drug encapsulation and release were observed when the PVP block length was increased from 86 to 235 RUs. With a PVP block length of 235 RUs, the micelles exhibited differing encapsulation and release properties for each drug. Minimal release was observed for doxorubicin (10%, pH 4.5) and CDKI-73 exhibited moderate release (77%, pH 4.5), whereas gossypol exhibited the best combination of encapsulation efficiency (83%) and release (91% pH 4.5) overall. These data demonstrate the drug selectivity of the PVP core, where both the block molecular weight and hydrophobicity of the core (and accordingly the hydrophobicity of the drug) have a significant effect on drug encapsulation and release. These systems remain a promising means of achieving targeted, pH-responsive drug delivery—albeit for select, compatible hydrophobic drugs—which warrants their further investigation to develop and evaluate clinically relevant micelle systems.

## 1. Introduction

The effective delivery of poorly bioavailable, hydrophobic drugs for anti-cancer treatments is a persistent challenge within the pharmaceutical sector. pH-responsive polymeric micelles have shown considerable promise as a means of achieving targeted and controlled delivery [1,2]. For site-specific drug release, block copolymers and their corresponding micelles can be engineered to exploit the pH differential existing between healthy tissues (pH 7.4) and the tumour milieu (pH ~6.5–7.2) or the intracellular organelles of cancerous cells (endo/lysosome pH 5.0–6.5) to trigger drug release [1,3,4]. Cancerous tumours can be passively and actively targeted by suitably sized micelles (<200 nm), following their accumulation at a target site via the enhanced permeation and retention (EPR) effect [5,6].

However, there are important caveats regarding the synthesis and application of common pH-responsive block copolymers. Syntheses are generally achieved via atom-transfer radical polymerisation (ATRP) or sequential living anionic polymerisation, which require multiple freeze–vacuum–thaw cycles [7,8,9] or low temperatures [10,11,12,13], respectively. In some cases, the relative complexity of such synthetic approaches can detract from the low dispersity (*Đ_avg_* ≈ 1.19) and control over block length, which both methods afford. There is also a dearth of information regarding the relationship between the molecular weight of the copolymer blocks—particularly the hydrophobic core—and how it governs the drug encapsulation and release properties of the resultant micelles. In addition, the suitability of both common and emerging hydrophobic anti-cancer drugs for use in these systems requires further investigation.

An example micelle system for which these caveats are evident is one prepared from diblock copolymers of PEG and poly(2- or (4-vinylpyridine) (PVP and P4VP, respectively). PEG is the most common hydrophilic corona-forming block used in the preparation of polymeric micelles, primarily due to its low toxicity and immunogenicity, and relatively low protein fouling, which enables micelles to evade the reticuloendothelial system (RES) and prolongs their circulation in situ [14,15]. PVP (or P4VP) as a core-forming block affords pH-responsive micelles [16], as the pH-dependent solubility of these blocks (pKa ~5) enables the triggering of micelle assembly and disassembly within high (pH > 5) and low (pH < 5) environments, respectively. In the past two decades, the size, shape, complexation, and pH-responsiveness of polymeric micelles prepared from PEG-*b*-PVP (or P4VP) copolymers have been investigated [7,8,9,10,11,12,13,15,17,18,19,20,21,22,23,24,25]. However, few studies have explored modern, facile synthetic methods for the preparation of the constitutive diblock copolymers, nor the effects of their composition on target drug suitability, and encapsulation and release.

For example, Iurciuc-Tincu et al. prepared PEG-*b*-PVP micelles loaded with curcumin and 5-fluorouracil, which were found to exhibit favourable bio- and hemocompatibility [11]. The diblock copolymers from which the micelles were prepared, were synthesised via anionic polymerisation [11,17]. Like syntheses conducted via ATRP for diblock copolymers containing P4VP [7,9], both synthetic approaches require complex deoxygenation and temperature-control, and could be replaced by comparatively facile and modern polymerisation techniques. The micelles were loaded with curcumin and 5-fluorouracil with good drug encapsulation efficiency (70% and 64%, respectively), and were found to exhibit pH-dependent release characteristics. At acidic pH (2.0), both micelles released ~90% of the encapsulated drug after 150 h. However, in both cases moderate release occurred at pH 7.4 (~60% and ~40% for curcumin and 5-fluorouracil, respectively) within a similar period. In the context of anti-cancer treatment—particularly for 5-fluorouracil—the significant drug release that occurred at pH 7.4 suggested poor targeting of release to the endo/lysosomal environments (pH 5.0–6.5), as the premature release of 5-fluorouracil within healthy tissues is likely. The authors attributed the slower release of curcumin (cf., 5-fluorouracil) to its greater hydrophobicity and stronger hydrophobic interactions with the PVP block. The same explanation is plausible for the poor pH-targeted release of micelles loaded with 5-fluorouracil and highlights the importance of investigating the effects of the PVP core—in combination with drugs with different physiochemical properties—on drug encapsulation and release.

Herein, we sought to further understand the effect of the block length of the PVP core (and hence the weight fraction of PVP (*f_PVP_*)) on the encapsulation and release of hydrophobic drugs within a PEG-*b*-PVP polymeric micelle system. A previously reported and facile, visible light-mediated photocontrolled reversible addition-fragmentation chain-transfer (photo-RAFT) polymerisation method [26] was used to synthesise PEG-*b*-PVP diblock copolymers, with narrow *Đ*. For the first time, PEG-b-PVP micelles were used to investigate the encapsulation and release of three hydrophobic drugs: gossypol, doxorubicin, and cyclin-dependent kinase inhibitor 73 (CDKI-73), with varying PVP block lengths of the constitutive pH-responsive copolymers. This work demonstrates the applicability of the photo-RAFT approach for the downstream preparation of micelles and provides valuable insight into the compatibility of hydrophobic drugs with PEG-*b*-PVP micelles, and the structural features governing this compatibility.

## 2. Experimental

### 2.1. Materials

All materials were used as received unless otherwise specified. 2-Vinylpyridine (VP; 97%) was purchased from Sigma Aldrich (St. Louis, MO, USA) and passed through basic aluminium oxide to remove inhibitors immediately prior to use. Basic aluminium oxide, triethanolamine (TEOA; >99%), 2-(dodecylthiocarbonothioylthio)-2-methylpropionic acid (DDMAT; 98%), *N*,*N*’-dicyclohexylcarbodiimide (DCC; 99%), 4-dimethylaminopyridine (DMAP; >99%), deuterium oxide (D_2_O; 99.9 atom% D), deuterium chloride (DCl; 35 wt% in D_2_O, ≥99 atom% D), deuterated chloroform (CDCl_3_; 99.9 atom% D), and phosphate buffered saline (PBS) tablets were purchased from Sigma Aldrich (St. Louis, MO, USA). α-Methoxyl-ω-hydroxy poly(ethylene glycol) (PEG; Tokyo Chemical Industry (TCI), average molecular weight 3.8 to 4.3 kDa), analytical reagent grade sodium citrate dihydrate, citric acid, dimethyl sulfoxide (DMSO), tetrahydrofuran (THF), diethyl ether (DEE), hexanes (Hex), dichloromethane (DCM), methanol (MeOH), ethanol (EtOH; 100% undenatured), hydrochloric acid (HCl, 36.5–38.0%), and acetone, were purchased from ChemSupply (Gillman, Australia). Cyclin-dependent kinase inhibitor 73 (CDKI-73) and doxorubicin hydrochloride were purchased from SelleckChem (Houston, TX, USA). Gossypol acetic acid was purchased from Shaanxi Ciyuan Biotech (Xi’an, China). Snakeskin^®^ dialysis tubing (7 kDa, and 1 kDa molecular weight cut-off (MWCO)) were purchased from Progen Biosciences (Archerfield, Australia) and hydrated in PBS (pH 7.4) prior to use. Polylite^TM^ poly(lactic acid) (PLA) 3D printing filament (1.75 mm) (Polymaker, Shanghai, China) was purchased from 3D Printing Solutions (Para Hills, Australia). A 60 mm diameter cooling fan (12 V DC, 1.6 W) was purchased from Jaycar Electronics (Gepps Cross, Australia). A generic blue LED light strip (12 V, ~460 nm) was purchased from AliExpress and driven by a 12 V, 2 A power supply.

PBS (pH 7.4 and 6.5) solutions were prepared following the manufacturer’s guidelines, with final pH adjustments made via the addition of 0.1 M HCl. Deuterated PBS was prepared using a PBS tablet and D_2_O, following the manufacturer’s instructions for the preparation of non-deuterated PBS. Buffered solutions (pH 5.5 and 4.5) were prepared using a citric acid/sodium citrate buffer, prepared according to the United States Pharmacopeia (USP 29).

### 2.2. Characterisation

The composition and number-average molecular weights (*M_n_*) of the PEG*_x_*PVP*_y_* copolymers were determined via ^1^H NMR spectroscopy using a Bruker Avance III HD 500 NMR spectrometer (Billerica, MA, USA), operating at 500 MHz, and calculated using the ratios of the integral values of the characteristic resonances at δ_H_ 3.63 (PEG RU), 3.36 (PEG methoxy end-group), and 8.20 (PVP RU) ppm.

The molecular weight characteristics of the polymers, including *M_n_,* weight-average molecular weight (*M_w_*) and *Ð* were determined via gel permeation chromatography (GPC), using a Shimadzu Prominence liquid chromatography system (Kyoto, Japan) fitted with a refractive index detector (Shimadzu, RID-20A), two mixed gel columns in series (Shimadzu GPC-80MD and GPC-804D), and using THF as the mobile phase (1 mL·min^−1^, 40 °C). A conventional column calibration was performed with a series of narrow molecular weight polystyrene standards (Polymer Standards Service GmbH, Mainz, Germany).

Copolymer syntheses were monitored, and monomer conversion was determined via gas chromatography (Shimadzu GC-2010 fitted with a flame ionisation detector (FID)), based on pre-polymerisation concentrations, using the solvent (DMSO or MeOH) as an internal reference. Aliquots of the crude reaction mixture were diluted in DCM (~1.5 mL) prior to analysis and introduced using a split injection (50:1 ratio) to a Supelco SPB-35 stationary phase column (30.0 m × 0.25 mm, 0.25 µm). The GC was operated in linear velocity mode with nitrogen (BOC, 99.999%) as the carrier gas (56.5 cm·s^−1^), with injection port and FID temperatures of 250 °C, and a temperature program of 35 °C (1 min hold) to 185 °C (1 min hold) at 30.0 °C·min^−1^.

Particle size distributions were measured via dynamic light scattering using a Malvern Zetasizer Nano ZS (He-Ne laser, λ = 633 nm, 4 mW) (Malvern, United Kingdom), using a cell temperature of 25 °C.

Drug concentrations from release experiments were quantified via UV-visible spectrophotometry using a Thermo Fisher Evolution 260 Bio (Waltham, MA, USA), fitted with a Thermo Fisher Peltier Control Cooling Unit, and operating at a cell temperature of 25 °C). Absorbances were determined at the lambda maximum for each drug (CDKI-73 = 275 nm, doxorubicin = 494 nm, and gossypol = 386 nm). Standard curves were generated across the concentration range of 0.5 to 12 µg·mL^−1^, and drug concentrations were quantified using molar absorption coefficients (ε) determined in each buffered solution (pH 7.4–4.5), for each drug (supporting information (SI), Table S1).

The pH values of the solutions were measured throughout using an Oakton pH 700 pH meter (Environmental Express, Charleston, SC, USA) fitted with an Oakton All-in-One pH/ATC Probe.

### 2.3. Procedures

#### 2.3.1. Synthesis of α-Methyl-ω-2-(dodecylthiocarbonothioylthio)-2-methylpropionate Poly(ethylene glycol) (PEG-DDMAT)

PEG (2.00 g, 500 µmol), DDMAT (275 mg, 754 µmol), DCC (155 mg, 751 µmol), DMAP (3.1 mg, 25.4 µmol), and DCM (10 mL) were combined in a sealed vial and stirred at ambient temperature (23 °C) for 72 h in the dark. The supernatant of the crude reaction mixture was isolated via centrifugation (6 krpm, 3 min), and the solvent was removed in vacuo (40 °C, 10 mbar). The crude product was redissolved in MeOH (5 mL) and dialysed (1 kDa MWCO) against EtOH (500 mL) for 72 h. The solution was concentrated in vacuo (40 °C, 10 mbar) and the residue was dried (23 °C, 0.02 mbar) to afford PEG-DDMAT as a yellow solid. GPC (THF) *M_n_* = 4.86 kDa, *M_w_* = 4.91 kDa, *Ð* = 1.01. ^1^H NMR (500 MHz, CDCl_3_, 23 °C) δ_H_ 4.24 (*t*, CH_2_OCO, end-group), 3.63 (*s*, CH_2_O, PEG RU), 3.55–3.52 (*m*, CH_2_O, end-group), 3.37 (*s*, CH_3_O, end-group), 3.25 (*t*, CH_2_S, end-group), 1.69 (*s*, CH_3_, end-group), 1.65 (*q*, CH_2_, end-group), 1.25 (*br s*, CH_2_, end-group), 0.87 (*t*, CH_3_, end-group) ppm. GPC chromatograms and ^1^H NMR spectra are provided in the Supplementary Materials, Figures S1 and S2, respectively. 

#### 2.3.2. Synthesis of Poly(ethylene glycol)_x_-b-poly(2-vinylpyridine)_y_ (PEG*_x_*PVP*_y_*) Copolymers

The synthetic method was adapted from a procedure reported by Fu et al. [26]. Inhibitor-free VP (821 µL, 7.61 mmol), PEG-DDMAT (73.9 mg, 15.2 µmol), and TEOA (21.0 uL, 158 µmol) were combined in MeOH (PEG_90_PVP_46_, PEG_90_PVP_58_, and PEG_90_PVP_86_) or DMSO (PEG_90_PVP_152_ and PEG_90_PVP_235_) (410.3 µL, 50 vol% cf. monomer) in a sealed vial (VP:PEG-DDMAT:TEOA, 500:1:7 mol equiv.). The vial was placed inside of a custom 3D-printed reaction chamber containing a blue LED light source (460 nm) (Supplementary Materials, Figures S3 and S4). The start of the reaction was marked by switching ‘on’ the light source, and the reaction was stopped by switching ‘off’ the light source after 6, 9, 12, 24, or 48 h; an aliquot was removed for GC analysis, which revealed monomer conversions of 6.9, 10.0, 15.2, 33.4, and 41.4%, respectively. The polymer product was immediately precipitated from a solution of diethyl ether:hexanes (1:1 *v*/*v*, 10 mL, ~4 °C) and isolated via centrifugation (12 krpm, 3 min). The precipitate was redissolved in DCM (1.5 mL) and this process was repeated for a total of three precipitations. The precipitate was dried in vacuo (0.02 mbar, 23 °C) to afford the copolymers as waxy, yellow solids. GPC: PEG_90_PVP_46_ *M_n_* = 6.0 kDa, *M_w_* = 6.8 kDa, *Ð* = 1.13; PEG_90_PVP_58_ *M_n_* = 6.3 kDa, *M_w_* = 7.5 kDa, *Ð* = 1.19; PEG_90_PVP_86_ *M_n_* = 10.6 kDa, *M_w_* = 12.1 kDa, *Ð* = 1.14; PEG_90_PVP_152_ *M_n_* = 14.1 kDa, *M_w_* = 17.3 kDa, *Ð* = 1.23; PEG_90_PVP_235_ *M_n_* = 21.4 kDa, *M_w_* = 25.8 kDa, *Ð* = 1.21. Representative ^1^H NMR (500 MHz, CDCl_3_, 23 °C) δ_H_ 8.48–8.05 (*m*, CH, PVP RU), 7.34–7.02 (*m*, CH_,_ PVP RU), 7.02–6.59 (*m,* CH, PVP RU), 6.53–6.12 (*m,* CH, PVP RU), 3.63 (*s*, CH_2_O, PEG RU), 3.36 (*s*, CH_3_O, PEG methoxy end-group), 2.30–1.30 (*m*, CH_2_S CTA end-group and CH_2_CH PVP RU), 1.24 (*br s*, CH_2_, CTA end-group), 0.87 (*t,* CH_3_ CTA methyl end-group) ppm. GPC chromatograms and ^1^H NMR spectra are provided in the Supplementary Materials, Figures S1 and S5, respectively.

#### 2.3.3. Polymeric Micelle Preparation

Polymeric micelles were prepared from the PEG*_x_*PVP*_y_* copolymers via the co-solvent evaporation approach. An equal volume of PBS (10 mM, pH 7.4) was added dropwise to the copolymer solution (1 mg·mL^−1^, acetone) under sonication. The mixture was then heated at 60 °C until the acetone had evaporated to afford a 1 mg·mL^−1^ micellar solution. The resultant solutions were pH-adjusted via the addition of small volumes of 0.1 M HCl to afford micellar solutions of ~1 mg·mL^−1^ at pH 4.0–7.4.

#### 2.3.4. ^1^H NMR Spectroscopy of Polymeric Micelles 

Polymeric micelles were prepared from PEG_90_PVP_235_ as described above at pH 7.4, except for the use of deuterated PBS (pH* 7.44), at a concentration of 1 mg·mL^−1^. The sample was analysed via ^1^H NMR spectroscopy (D_2_O, 500 MHz, 23 °C), then adjusted to pH* 4.53 via the addition of DCl in D_2_O (0.1 M) and re-analysed, where pH* values are pH measurements recorded in D_2_O using a H_2_O-calibrated pH meter (pH = 0.929pH* + 0.41) [27].

#### 2.3.5. Drug Encapsulation and Release

Copolymers (PEG_90_PVP_235_ or PEG_90_PVP_86_) were solubilised in acetone with the target drug (CDKI-73, doxorubicin, or gossypol) to afford concentrations of 0.9 and 0.1 mg·mL^−1^, respectively. Polymeric micelles were then prepared as previously described to afford 1 mg·mL^−1^ drug-loaded micellar solutions in PBS (10 mM, pH 7.4) with a drug loading of 10 wt%. Drug-loaded micellar solutions (4 mL) were then placed in dialysis tubing (7 kDa MWCO) and suspended in a pH 7.4 receiving solution (40 mL, 15 h) to remove any unencapsulated drug. The receiving solution was then replaced with buffered solutions of decreasing pH every 3 h (pH 6.5, 5.5, and 4.5) to investigate the release of the encapsulated drug. Aliquots of the receiving solution (1 mL) were removed hourly and analysed using UV-visible spectrophotometry to quantify drug release. The total volume of the receiving solution was maintained throughout the experiment via the addition of 1 mL of buffered solution at the appropriate pH.

## 3. Results and Discussion

**Scheme 1:**
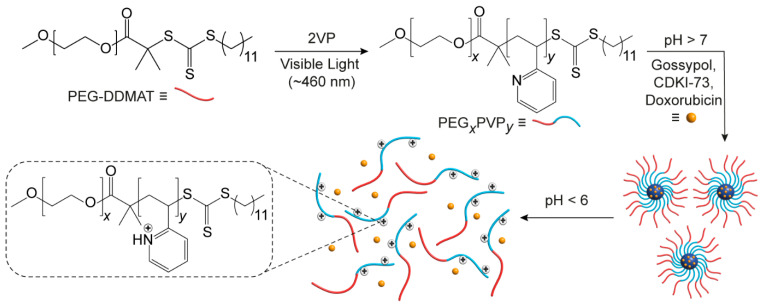
Preparation of PEG-*b*-PVP copolymers via the photo-RAFT of 2-vinylpyridine from the PEG-DDMAT macroinitiator, and the pH-mediated formation and disassembly of their drug-loaded micelles.

Using a trithiocarbonate RAFT macroinitiator (PEG-DDMAT) (Supplementary Materials, Figure S2), a series of linear diblock PEG-*b*-PVP copolymers were synthesised via visible-light-mediated RAFT (photo-RAFT) polymerisation [26] using a 460 nm visible light source (Scheme 1). The polymerisation was stopped after defined periods by switching off the light source, allowing for control over the monomer conversion and the PVP block length (Table 1). As reported previously by Fu et al. for the photo-RAFT of acrylates, the inclusion of a sacrificial amine (triethanolamine) in combination with trithiocarbonate as a photoredox catalyst for deoxygenation and polymerisation, enabled the well-controlled, oxygen-tolerant photo-RAFT of vinylarenes (i.e., vinylpyridine) from trithiocarbonate macroinitiators.

A series of copolymers were prepared with a common PEG-DDMAT initiator (90 RU, *M_n_* = 4.3 kDa) to allow the effect of the PVP block length on micelle hydrodynamic diameter (*D_h_*), pH-responsiveness, and drug encapsulation and release to be investigated. PEG-*b*-PVP copolymers were synthesised with PVP block lengths and weight fractions of PVP (*f_PVP_*) ranging from 46–235 RU and 0.53–0.85, respectively (Table 1). The copolymers were referred to as PEG*_x_*PVP*_y_*, where *x* and *y* refers to the number of PEG and PVP RUs, respectively. ^1^H NMR spectroscopic analysis of the copolymers (Supplementary Materials, Figure S5) provided *M_n_* values consistent with the theoretical *M_n_* values determined from GC quantification of the monomer conversions. GPC of the copolymers provided *Đ* values of ≤1.23 for all copolymers (Supplementary Materials, Figure S1), indicating good control over the polymerisation.

**Figure 1:**
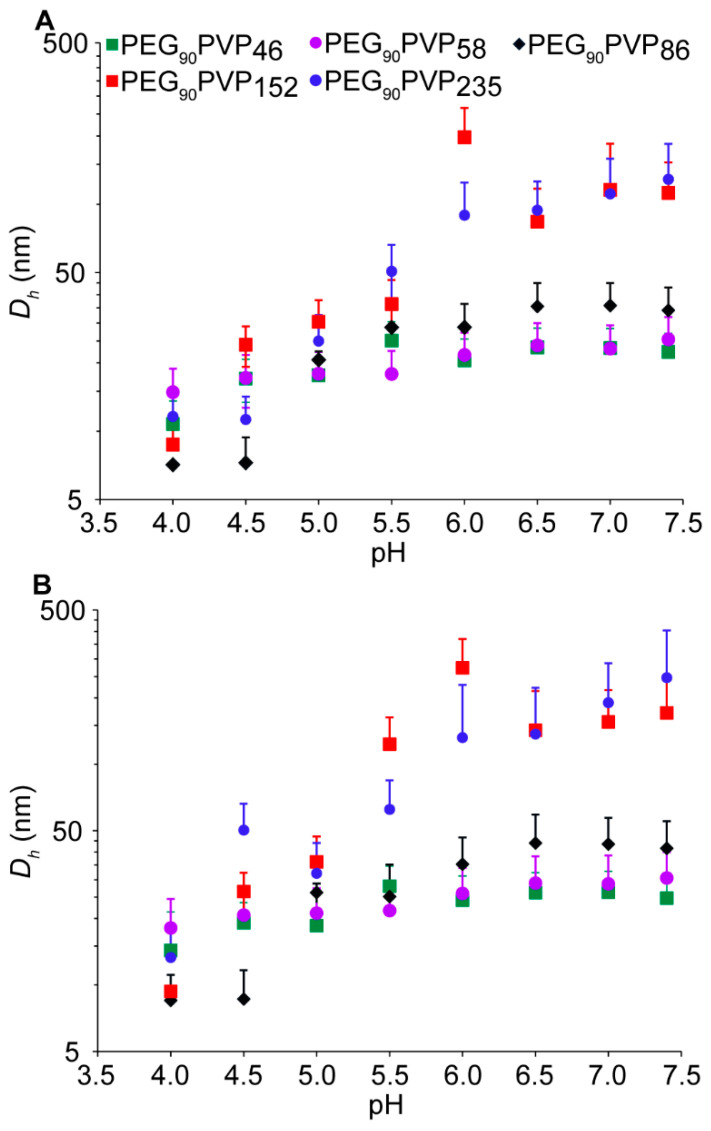
The (**A**) number and (**B**) volume average particle size distributions (PSDs) (Supplementary Materials, Figure S6 and Table S2). Values are reported as mean + std. dev.

Micelles were prepared in PBS (10 mM, pH 7.4, 1 mg·mL^−1^) from the copolymers using the co-solvent evaporation approach, then pH-adjusted to afford solutions with pH values ranging from 7.4 to 4.0. Particle size distributions (PSD) determined by DLS revealed the successful self-assembly of copolymers into micelles (Figure 1), which were stable for at least several days following preparation and storage at 23 °C. At physiological pH (7.4), the number-weighted distributions showed that the *D_h_* values of the micelles increased with increasing *f_PVP_* of the copolymers, which ranged from 22 nm (PEG_90_PVP_46_) to 130 nm (PEG_90_PVP_235_) (Supplementary Materials, Table S2), and generally exhibited low PDI values (Supplementary Materials, Table S3).

The *f_PVP_* of the copolymers were positively correlated with both the *D_h_* and PDI of the micelle populations at pH 7.4 (Figure 1 and Supplementary Materials, Tables S2 and S3). These trends are consistent with previous studies, whereby smaller, well-defined micelle morphologies were reported for PEG-*b*-PVP copolymers (prepared from both VP and 4-vinylpyridine (4VP)) in which the PEG block predominated, and comparatively poorly defined and larger micellar aggregates or vesicles formed when the PVP block predominated [10,12].

When the pH of the micellar solutions was decreased, a gradual decline in *D_h_* was observed at pH 6.0–5.5, which correlates to protonation/ionisation of the PVP block as the pH approaches the pK_a_ of VP (pK_a_ ~5) and previously reported pK_a_ values for PEG-*b*-PVP copolymers [12,13,17]. Protonation of the PVP block would be expected to cause micelle swelling, reorganisation, and disassembly, until unimers are observed at a sufficiently low pH. For the higher *f_PVP_* copolymers (i.e., PEG_90_PVP_152_ and PEG_90_PVP_235_), disassembly was preceded by a slight increase in *D_h_* at pH 6.0. This is likely due to the presence of partially protonated, and electrostatically repulsive PVP blocks at the perimeter of the micelle core, hindering the steric stabilisation afforded by the PEG blocks and causing swelling and an increase in micelle size. This observation is consistent with previously reported results for micelles containing PVP [7,12,13,17]. The same trend is not observed for copolymers with *f_PVP_* < 0.68 (i.e., PEG_90_PVP_46_, PEG_90_PVP_58_, and PEG_90_PVP_86_), ostensibly due to the greater stability conferred by PEG in these micelles where a larger weight fraction of PEG exists. Regardless, further reduction in pH (and protonation of the PVP blocks) afforded an apparent disassembly of the micelles into smaller aggregates or unimers for all the studied copolymers (Table S2).

**Figure 2:**
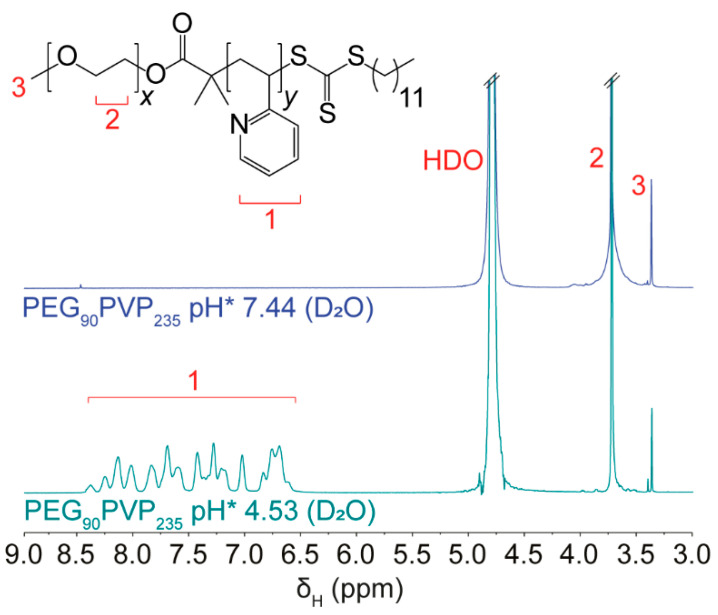
^1^H NMR spectra (D_2_O, 500 MHz, 23 °C) of PEG_90_PVP_235_ micelles prepared in deuterated PBS at pH* 7.44 (1 mg·mL^−1^) and then adjusted to pH* 4.53.

The DLS results of the micelle solutions were supported by ^1^H NMR spectroscopy, which revealed protonation of the PVP block at low pH. For example, the NMR spectrum of PEG_90_PVP_235_ in deuterated PBS (pH* 7.44, 1 mg·mL^−1^) revealed a resonance from the PEG block but no aromatic proton resonances (~8.5–6.5 ppm) from the neutral PVP block due to their poor solvation and restricted mobility within the tightly packed micelle core (Figure 2). However, when the pH* was adjusted to 4.53, the PVP proton resonances were visible, due to their protonation and the disruption of the micelle structure (caused by electrostatic repulsion between protonated pyridinyl moieties), resulting in increased solvation and greater mobility. These observations were consistent with those reported by Atanase et al. [17] and indicated the successful assembly and disassembly of the micelles through protonation of the PVP block.

The observed reorganisation and disassembly of the micelles between pH 6.0–5.5 implied that the micelles would be responsive to the endosomal environment [4], which could be manipulated as a trigger for the release of an encapsulated drug. To investigate this prospect and whether the physiochemical properties of therapeutics play a role in their triggered release from the micelles, we assessed the encapsulation and release of three hydrophobic drugs (Table 2) with the potential for cancer treatment, as a function of pH and *f_PVP_*.

Drug-loaded polymeric micelles (10 wt% drug loading) were initially prepared using the PEG_90_PVP_235_ copolymer in PBS at pH 7.4 and dialysed against a pH 7.4 receiving solution for 15 h to assess for the presence of any unencapsulated drug. The pH of the receiving solution was then decreased by ~1 pH unit, every 3 h, to pH 4.5, to simulate the low pH environments of the cancerous tumour milieu and intracellular organelles (i.e., the endo/lysosome) [33].

**Figure 3:**
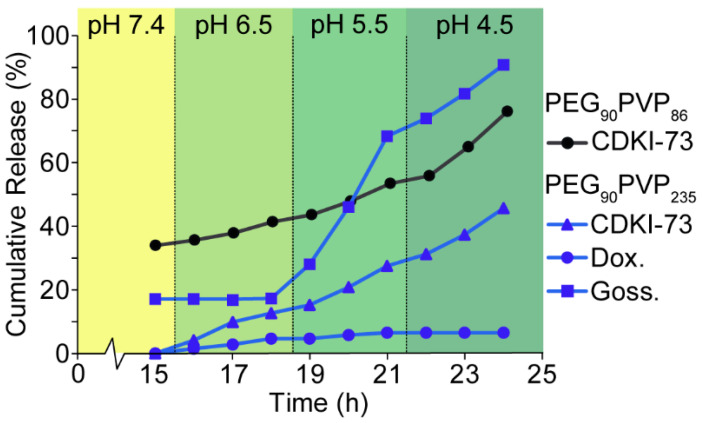
pH-dependent cumulative release plots for the PEG_90_PVP_86_ and PEG_90_PVP_235_ copolymers following the encapsulation of hydrophobic drugs CDKI-73, doxorubicin, or gossypol. Drug-loaded micellar solutions of concentration 1 mg·mL^−1^ were used for all experiments, with a drug loading of 10 wt%.

All three drugs were successfully encapsulated in the PEG_90_PVP_235_ micelles; however, a marked difference in encapsulation and release was observed (Figure 3). Gossypol showed an idealised release profile, with 83% drug encapsulation efficiency (17% unencapsulated drug release at pH 7.4) and rapid release starting at pH 5.5 and reaching a maximum of 91% at pH 4.5, consistent with the pH-mediated disassembly of the polymeric micelles. In contrast, doxorubicin exhibited 100% encapsulation (no unencapsulated drug detected at pH 7.4) but failed to release a significant amount of drug (<10% at pH 4.5). This difference was attributed to the hydrophobicity of both drugs and their non-covalent interactions with the PVP block. As gossypol is more hydrophobic compared to doxorubicin (Table 2), it is conceivable that the van der Waals forces present between gossypol and the micelle core are disrupted through protonation of the PVP blocks at low pH (≤5.5). Whereas, at higher pH values (≥6.5), van der Waals forces and H-bonding between the phenolic groups of gossypol and the PVP core would contribute to its retention. The increase in core hydrophilicity upon protonation of the PVP blocks likely results in disruption of these non-covalent interactions causing micelle disassembly and the release of gossypol into the receiving solution.

Contrastingly, the more polar doxorubicin was likely retained due to the presence of π–π stacking with the PVP blocks, which could be attributed to the planar anthraquinone motif of doxorubicin (cf., gossypol), which is also responsible for the intercalation of doxorubicin with DNA [34,35]. At all tested solution pH values, the amine group of doxorubicin would be protonated. The electrostatic repulsion afforded at low pH between the protonated doxorubicin and PVP blocks was likely insufficient to induce complete micelle disassembly due to π–π stacking. The small amount of release observed for PEG_90_PVP_235_ micelles containing doxorubicin may be due to the loss of loosely bound doxorubicin, whilst the majority was retained.

The encapsulation and release of CDKI-73 exhibited a comparatively intermediate profile, with 100% encapsulation efficiency (no release detected at pH 7.4) and an approximately linear release profile with decreasing pH, reaching an overall release of 46%. However, a lack of available data makes it challenging to relate these results to the physicochemical properties of CDKI-73. An estimated LogP value of 2.92 for CDKI-73 (Table 2) suggests that these results are consistent with its intermediate hydrophobicity (cf. doxorubicin and gossypol). Of the various functional groups in CDKI-73, only the 5-aryl-2-aminothiazole motif might be expected to have a pKa value that is of significance in this study. If it is assumed that CDKI-73 has a similar pKa value to the structural analogue, 5-phenyl-2-aminothiazole (pKa = 4.9) [36], then CDKI-73 would become protonated and positively charged in parallel with PVP protonation/ionisation. This ionisation would cause electrostatic repulsion between CDKI-73 and the PVP blocks, driving micelle disassembly and drug release in the absence of other sufficiently strong non-covalent interactions (cf. doxorubicin). The observed trends for the three drugs indicated that very hydrophobic drugs are released rapidly upon a decrease in pH due to repulsion from the hydrophilic, protonated PVP blocks, whereas more polar drugs have a stronger attraction to the protonated PVP blocks which can impede release.

To assess the effect of the *f_PVP_* on encapsulation and release, CDKI-73-loaded micelles were prepared from PEG_90_PVP_86_ exhibiting a shorter PVP block length. A significant decrease in encapsulation efficiency (66%) was observed for the PEG_90_PVP_86_ micelles (cf. PEG_90_PVP_235_ micelles) (Figure 3), most likely because of the smaller *f_PVP_* not being sufficient to accommodate all the CDKI-73 at a loading of 10 wt%. The release profile of CDKI-73 from the PEG_90_PVP_86_ micelles with decreasing pH, followed a similar trend to that observed for the PEG_90_PVP_235_ micelles, with a total release of 77% at pH 4.5. This again suggested that the pH-dependent change in hydrophobicity—from hydrophobic to hydrophilic at low pH—and electrostatic repulsion between the protonated PVP blocks and protonated CDKI-73 facilitated drug release. When considering the unencapsulated portion of the drug, the pH-triggered release of encapsulated CDKI-73 from PEG_90_PVP_86_ micelles was 43%, comparable with the 46% release from PEG_90_PVP_235_ micelles. Doxorubicin was also successfully encapsulated in PEG_90_PVP_86_ micelles (encapsulation efficiency = 100%), but no release was observed within any of the tested receiving solutions. It is likely that the observed absence of doxorubicin release was again due to the relatively strong interactions between doxorubicin and the PVP micelle core. The reduction in encapsulation efficiency and similar release profile for the PEG_90_PVP_86_ polymeric micelles suggested that the *f_PVP_* predominately contributed to the loading capacity, while the underlying encapsulation and release mechanisms appeared unchanged.

## 4. Conclusions

A series of diblock PEG*_x_*PVP*_y_* copolymers were synthesised via photo-RAFT polymerisation, with varying *f_PVP_*. The PEG*_x_*PVP*_y_* copolymers exhibited pH-dependent micellisation, with *D_h_* values dependent on the *f_PVP_*. This pH-dependence is consistent with the protonation of the PVP block (pKa ~5), and electrostatic repulsion induced micelle disassembly. Gossypol, CDKI-73, and doxorubicin were successfully encapsulated with >83% efficiency in PEG_90_PVP_235_ micelles (10 wt% drug loading) at pH 7.4. Gossypol and CDKI-73 both exhibited favourable release profiles, with 91% and 46% released, respectively, over 9 h as the pH was decreased. In both cases, encapsulation was attributed to the hydrophobicity of the drugs and secondary interactions with the neutral PVP blocks at pH 7.4. Gossypol and CDKI-73 release at low pH was likely induced by repulsive interactions between the drugs and protonated PVP blocks and micelle disassembly. While a similar trend for CDKI-73 release was observed when encapsulated in PEG_90_PVP_86_ micelles, a reduction in encapsulation efficiency indicated that the loading capacity was dependent on the *f_PVP_*. The comparatively polar doxorubicin was almost completely retained in PEG_90_PVP_86_ and PEG_90_PVP_235_ micelles at low pH, likely due to the presence of π–π stacking interactions with the protonated PVP blocks. These data exemplify the drug selectivity of the PVP core in PEG-*b*-PVP polymeric micelles, whereby the *f_PVP_* and the hydrophobicity and physiochemical properties of the drug all have a significant effect on drug encapsulation and release. This highlights the need to evaluate target drugs for their compatibility with selected pH-responsive micelle systems. Furthermore, this study indicates the potential suitability of the PEG-*b*-PVP system for the triggered endosomal delivery of gossypol and CDKI-73, but not the more polar doxorubicin. In addition, the micelles were appropriately sized (<130 nm) to exploit the EPR effect for uptake into cancerous tumours. Together, these data show that PEG-*b*-PVP polymeric micelles remain a promising means of targeted, pH-responsive drug delivery—albeit for select, compatible hydrophobic drugs—and warrant further investigation to develop and evaluate clinically relevant micelle systems.

## Figures and Tables

**Table 1:** Polymerisation time, monomer conversion, and molecular weight characteristics of the synthesised PEG-*b*-PVP copolymers.

Copolymer Code ^a^	Polymerisation Time (h)	Theoretical *M_n_* (kDa) ^b^	Copolymer *M_n_*_,NMR_ (kDa) ^c^	PVP *M_n_*_,NMR_ (kDa) ^c^	Weight Fraction of PVP (*f_PVP_*) ^d^	Copolymer *M_n_*_,GPC_ (kDa) ^e^	*Đ* ^e^
PEG_90_PVP_46_	6	8.5	8.8	4.8	0.53	6.0	1.13
PEG_90_PVP_58_	9	10.1	10.7	6.1	0.58	6.3	1.19
PEG_90_PVP_86_	12	12.9	13.0	9.1	0.68	10.6	1.14
PEG_90_PVP_152_	24	22.4	20.0	16.0	0.79	14.1	1.23
PEG_90_PVP_235_	48	26.7	28.0	24.7	0.85	21.4	1.21

^a^ PEG-*b*-PVP copolymers were referred to as PEG*_x_*PVP*_y_*, where *x* and *y* indicate the number of PEG and PVP RUs, respectively. ^b^ *M_n_* (theoretical) based on PEG-CTA molecular weight and GC conversion of 2VP. ^c^ *M_n_* determined from ^1^H NMR spectroscopy end-group analysis. ^d^ Weight fraction of PVP = *M_n_* PVP/*M_n_* PEG-*b*-PVP copolymer. ^e^ Determined from GPC using a conventional column calibration with narrow molecular weight polystyrene standards.

**Table 2:** Structures and summary of the physicochemical properties of the encapsulated hydrophobic drugs.

Drug	Chemical Structure	LogP	LogS	pKa Value (s)
Gossypol	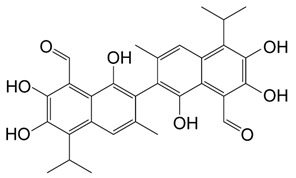	5–6.2 ^a^, 4.49 ^b^	5.13 ^b^	6.73 (1), ^d^ 7.38 (2), ^d^ 11.82 (3), ^d^ 12.68 (4), ^d^ 13.89 (5) ^d^
CDKI-73	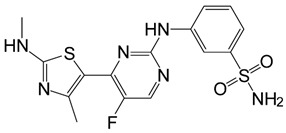	2.92 ^b^	4.19 ^b^	-
Doxorubicin	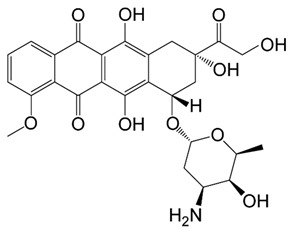	1.3 ^c^, 1.41 ^b^	2.67 ^b^	9.93 ^d^8.3 ^b^7.34 (phenol), ^d^ 8.46 (amine), ^d^ 9.46 ^d^

^a^ Obtained from Liu et al. [28] and Dodou et al. [29]. ^b^ Predicted using ALOGPS 2.1 as detailed by Tetko et al. [30] ^c^ Obtained from Alrushaid et al. [31] ^d^ Predicted using SPARC Ver 4.2. [32].

## Data Availability

The data presented in this study are available on request from the corresponding author.

## Supplementary Materials

The following supporting information can be downloaded at: https://www.mdpi.com/article/10.3390/pharmaceutics15030977/s1. Table S1: Summary of drug standard curve results; Figure S1: Summary of GPC chromatograms of the PEG-DDMAT initiator and PEG*_x_*PVP*_y_* copolymers; Figure S2: ^1^H NMR spectrum of PEG-DDMAT; Figure S3: Digital images of the 3D-printed reaction chamber; Figure S4: CAD models and drawings of the 3D-printed reaction chamber; Figure S5: ^1^H NMR spectra of the PEG*_x_*PVP*_y_* copolymers; Table S2: Summary of the *D_h_* values of the PEG*_x_*PVP*_y_* micelles; Table S3: Summary of the polydispersity index values of the PEG*_x_*PVP*_y_* micelles; Figure S6: DLS particle size distributions of the PEG*_x_*PVP*_y_* micelles.
