# Supplementary material for: pH-Responsive Poly(ethylene glycol)-b-poly(2-vinylpyridine) Micelles for the Triggered Release of Therapeutics
Source: Pharmaceutics. 2023 Mar 18;15(3):977. doi: 10.3390/pharmaceutics15030977 (PMC10056943; doi:10.3390/pharmaceutics15030977)
Supplement: Supplementary file 1 [file pharmaceutics-15-00977-s001.zip › pharmaceutics-2232055-supplementary.pdf]

# Supplementary Information

## pH-responsive Poly(ethylene glycol)-*b*-poly(2-vinylpyridine) Micelles for the Triggered Release of Therapeutics

Kyle Brewer, Fengxiang Bai, Anton Blencowe*

*Applied Chemistry and Translational Biomaterials (ACTB) Group, Centre for Pharmaceutical Innovation (CPI), UniSA Clinical and Health Sciences, University of South Australia, Adelaide, SA, 5000, Australia*

*Corresponding Author: anton.blencowe@unisa.edu.au

**Table S1:** Summary of the standard curve results for CDKI-73, doxorubicin, and gossypol, prepared in buffered solutions of pH 7.4–4.5.

| | pH | | | | | | | |
|---|---|---|---|---|---|---|---|---|
| | 7.4 | | 6.5 | | 5.5 | | 4.5 | |
| **Drug** | **$R^2$ Value** | **ε ($L.mol^{-1}.cm^{-1}$)[a]** | **$R^2$ Value** | **ε ($L.mol^{-1}.cm^{-1}$)[a]** | **$R^2$ Value** | **ε ($L.mol^{-1}.cm^{-1}$)[a]** | **$R^2$ Value** | **ε ($L.mol^{-1}.cm^{-1}$)[a]** |
| **CDKI-73** | 0.994 | 13864 ± 2060 (14.9) | 0.999 | 14294 ± 1391 (9.7) | 0.999 | 13844 ± 1002 (7.2) | 0.989 | 6553 ± 1825 (27.9) |
| **Doxorubicin** | 0.998 | 11403 ± 1214 (10.6) | 1.000 | 11665 ± 1587 (13.6) | 1.000 | 12129 ± 2147 (17.7) | 0.998 | 11357 ± 2039 (18.0) |
| **Gossypol** | 0.999 | 18325 ± 2171 (11.8) | 0.999 | 26651 ± 3509 (13.2) | 0.986 | 26904 ± 6571 (24.4) | 1.000 | 29814 ± 7065 (23.7) |

[a] Molar absorption coefficients are reported as mean ± std. dev. (percentage relative standard deviation), calculated from n = 6 absorbance measurements obtained at concentrations from 0.5–12 $\mu g.mL^{-1}$.

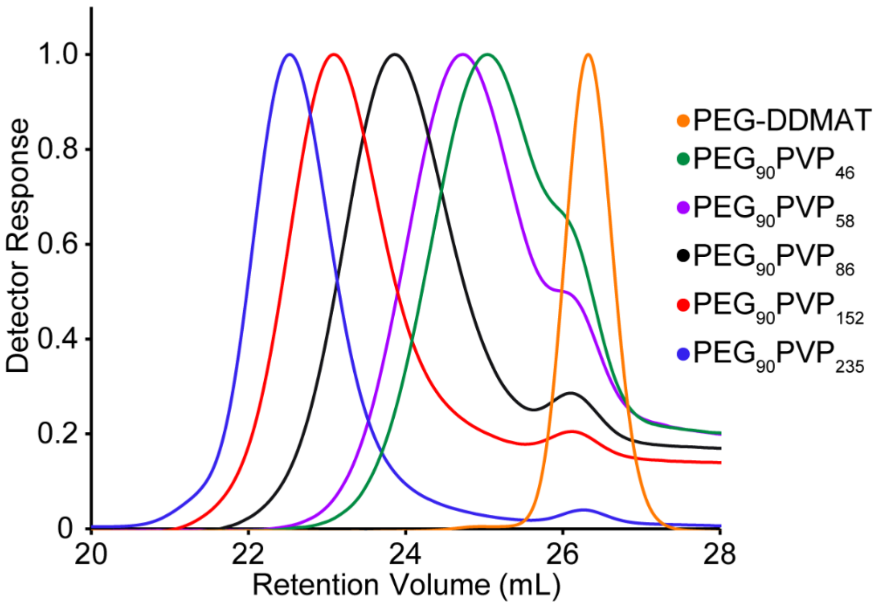


**Figure S1:** GPC differential refractive index chromatograms of the PEG-DDMAT macroinitiator and the PEG$_x$PVP$_y$ copolymers.

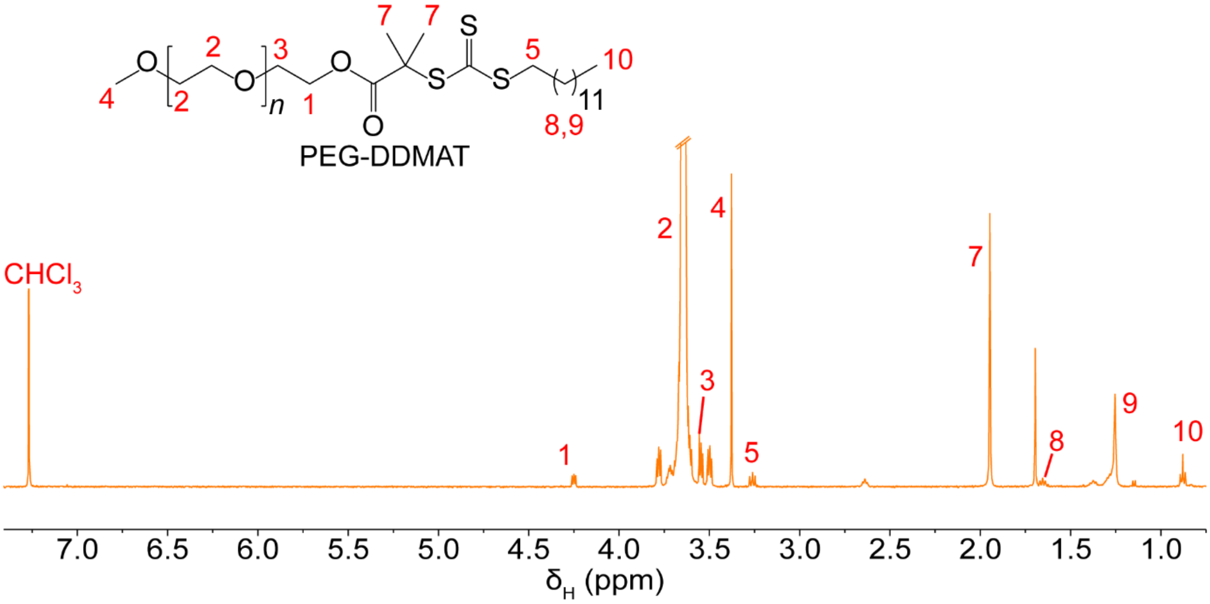


**Figure S2:** $^1$H NMR spectrum ($CDCl_3$, 500 MHz, 23 °C) of PEG-DDMAT macroinitiator.

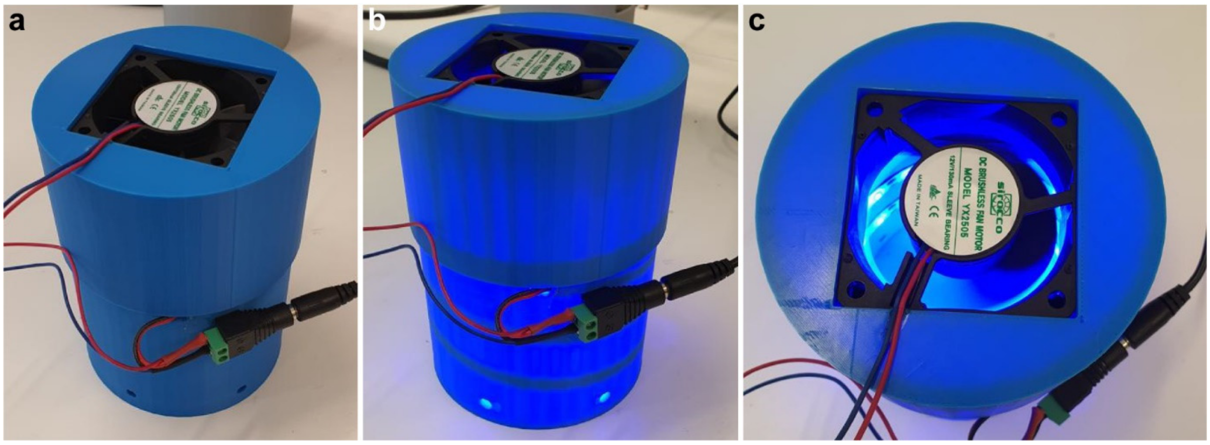


**Figure S3:** Digital images showing the 3D-printed reaction chamber used to conduct the photo-RAFT syntheses, whilst a) ‘off’, or b) ‘on’, and c) showing the fan used to regulate the internal temperature of the reaction chamber.

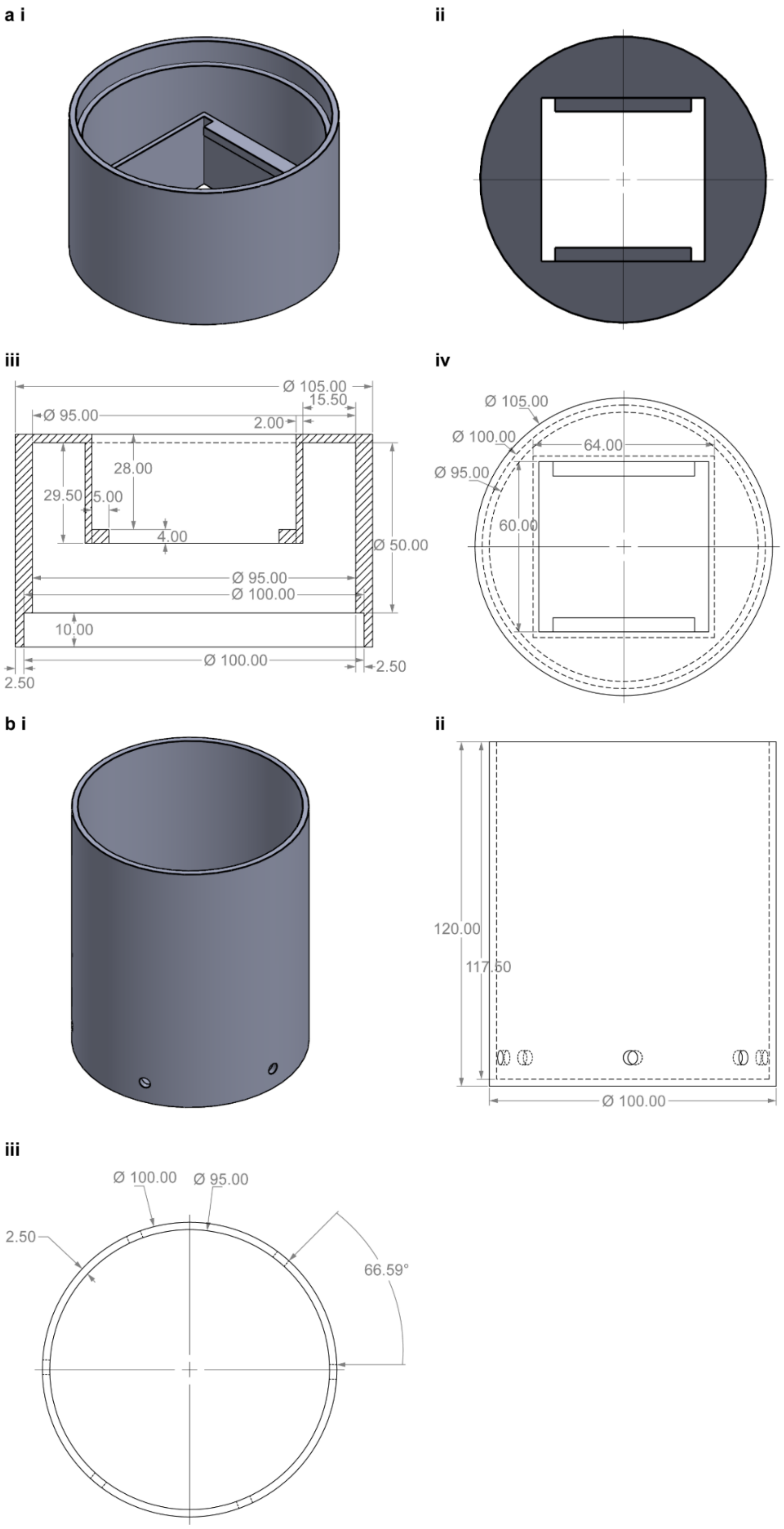


**Figure S4.** CAD models and drawings (Solidworks) of the 3D-printed reaction chamber showing a) the upper housing in which the cooling fan used to regulate the internal temperature of the reaction chamber was installed, and b) the lower housing in which the blue LED light source (as an LED strip) was affixed interiorly.

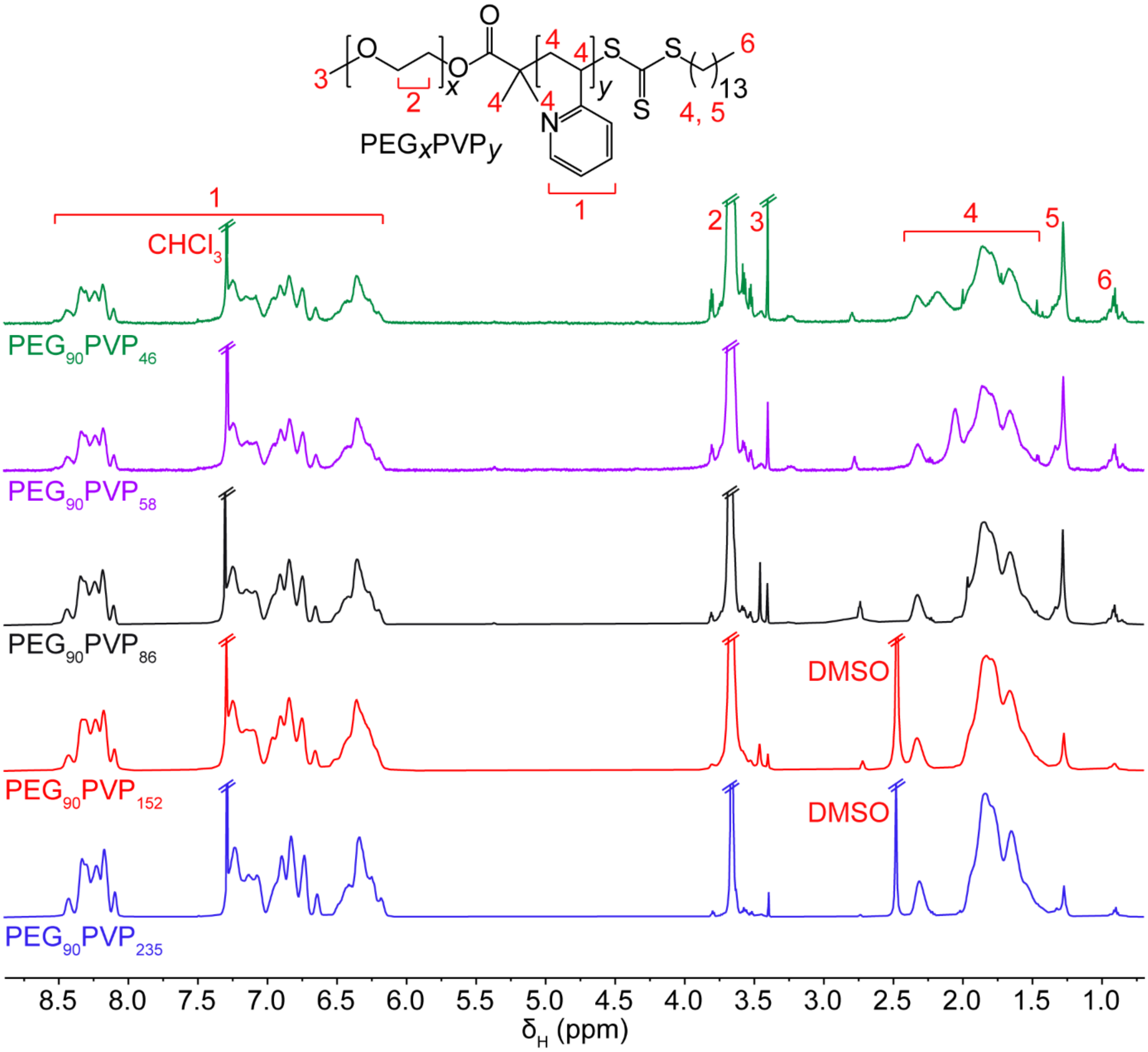


**Figure S5:** $^1$H NMR spectra ($CDCl_3$, 500 MHz, 23 °C) of the PEG$_x$PVP$_y$ copolymers with varying PVP block length.

**Table S2:** Summary of the hydrodynamic diameters ($D_h$) of polymeric micelles prepared from the $PEG_xPVP_y$ copolymers, obtained from the number- and volume-weighted distributions of the DLS analyses. Values are reported as the mean ± std. dev. of the peak size.

| | **Number-weighted Particle Size Distributions** | | | | |
|---|---|---|---|---|---|
| | **Diameter ($D_h$, nm)** | | | | |
| **pH** | $PEG_{90}PVP_{46}$ | $PEG_{90}PVP_{58}$ | $PEG_{90}PVP_{86}$ | $PEG_{90}PVP_{152}$ | $PEG_{90}PVP_{235}$ |
| **7.4** | 22.4 ± 4.6 | 25.4 ± 6.4 | 34.1 ± 8.9 | 112.3 ± 40.7 | 128.9 ± 55.6 |
| **7.0** | 23.3 ± 5.1 | 23.2 ± 6.2 | 35.8 ± 9.2 | 115.8 ± 69.2 | 111.4 ± 47.4 |
| **6.5** | 23.5 ± 5.0 | 23.9 ± 6.1 | 35.5 ± 9.4 | 83.8 ± 33.2 | 94.1 ± 31.8 |
| **6.0** | 20.5 ± 5.0 | 21.7 ± 5.5 | 28.8 ± 7.5 | 197.4 ± 68.4 | 89.5 ± 35.4 |
| **5.5** | 25.1 ± 5.2 | 17.9 ± 4.7 | 28.8 ± 7.5 | 36.4 ± 10.0 | 50.7 ± 15.6 |
| **5.0** | 17.7 ± 2.9 | 18.0 ± 4.4 | 20.7 ± 5.7 | 30.4 ± 7.5 | 25.0 ± 7.5 |
| **4.5** | 17.1 ± 3.7 | 17.2 ± 4.5 | 7.3 ± 1.9 | 24.1 ± 4.9 | 11.3 ± 2.9 |
| **4.0** | 10.8 ± 2.8 | 14.9 ± 4.0 | 7.1 ± 2.1 | 8.7 ± 1.5 | 11.6 ± 2.5 |
| | **Volume-weighted Particle Size Distributions** | | | | |
| **pH** | **Diameter ($D_h$, nm)** | | | | |
| **7.4** | 24.8 ± 5.6 | 30.5 ± 9.5 | 41.6 ± 13.5 | 171.0 ± 75.8 | 246.5 ± 158.0 |
| **7.0** | 26.3 ± 6.5 | 28.7 ± 9.9 | 43.5 ± 13.9 | 155.2 ± 61.5 | 190.0 ± 97.0 |
| **6.5** | 26.2 ± 6.1 | 28.9 ± 9.3 | 44.0 ± 15.2 | 142.5 ± 72.2 | 137.0 ± 85.0 |
| **6.0** | 24.2 ± 7.0 | 25.9 ± 8.1 | 35.2 ± 11.5 | 273.3 ± 95.8 | 132.0 ± 97.0 |
| **5.5** | 28.0 ± 6.6 | 21.7 ± 7.0 | 25.1 ± 11.5 | 123.3 ± 39.9 | 62.4 ± 22.2 |
| **5.0** | 18.6 ± 3.2 | 21.1 ± 6.3 | 26.2 ± 10.1 | 36.1 ± 11.0 | 32.0 ± 12.0 |
| **4.5** | 19.1 ± 4.5 | 20.7 ± 6.8 | 8.6 ± 2.6 | 26.5 ± 5.7 | 50.3 ± 15.9 |
| **4.0** | 14.4 ± 7.1 | 18.1 ± 6.4 | 8.5 ± 3.0 | 9.3 ± 7.8 | 13.3 ± 5.2 |

**Table S3:** Summary of the polydispersity index values of polymeric micelles prepared from each $PEG_xPVP_y$ copolymer, obtained *via* DLS.

| | Peak PDI | | | | |
|---|---|---|---|---|---|
| **pH** | $PEG_{90}PVP_{46}$ | $PEG_{90}PVP_{58}$ | $PEG_{90}PVP_{86}$ | $PEG_{90}PVP_{152}$ | $PEG_{90}PVP_{235}$ |
| **7.4** | 0.04 | 0.10 | 0.10 | 0.14 | 0.26 |
| **7.0** | 0.05 | 0.13 | 0.10 | 0.16 | 0.17 |
| **6.5** | 0.04 | 0.12 | 0.13 | 0.16 | 0.19 |
| **6.0** | 0.08 | 0.10 | 0.11 | 0.10 | 0.26 |
| **5.5** | 0.05 | 0.12 | 0.11 | 0.09 | 0.09 |
| **5.0** | 0.02 | 0.11 | 0.21 | 0.09 | 0.15 |
| **4.5** | 0.05 | 0.15 | 0.09 | 0.04 | 0.08 |
| **4.0** | 0.05 | 0.13 | 0.12 | 0.03 | 0.06 |

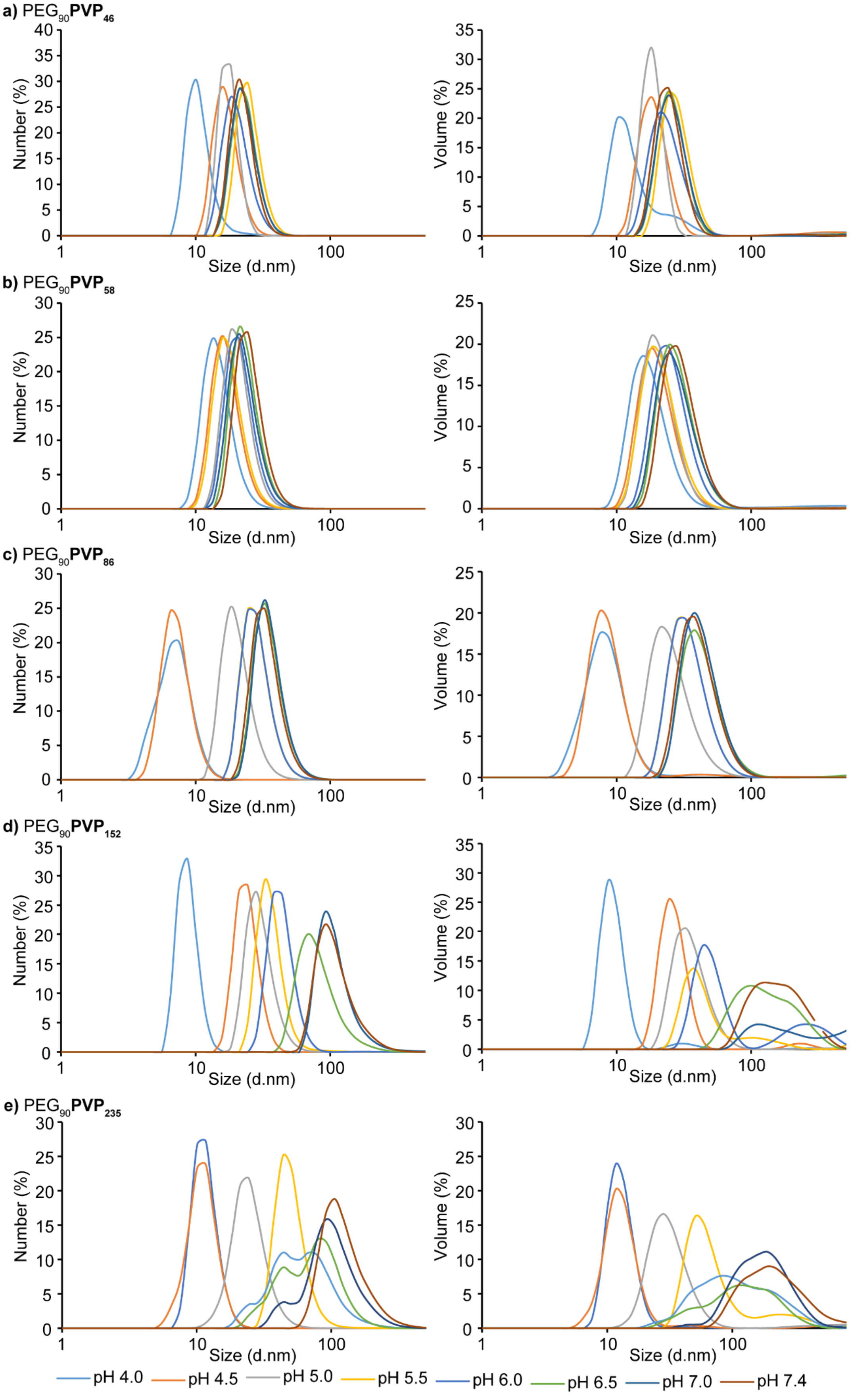


**Figure S6:** a)–e) DLS particle size distributions of the PEG$_x$PVP$_y$ (1 mg.mL$^{-1}$, 25 °C) copolymer micelles as a function of pH (7.4–4.0).
